# Evidence supporting deployment of next generation insecticide treated nets in Burkina Faso: bioassays with either chlorfenapyr or piperonyl butoxide increase mortality of pyrethroid-resistant *Anopheles gambiae*

**DOI:** 10.1186/s12936-021-03936-3

**Published:** 2021-10-18

**Authors:** Aristide S. Hien, Dieudonné D. Soma, Samina Maiga, Dramane Coulibaly, Abdoulaye Diabaté, Allison Belemvire, Mame B. Diouf, Djenam Jacob, Adama Koné, Ellen Dotson, Taiwo S. Awolola, Richard M. Oxborough, Roch K. Dabiré

**Affiliations:** 1 Institut de Recherche en Sciences de La Santé (IRSS), Bobo-Dioulasso, Burkina Faso; 2 U.S. President’s Malaria Initiative, U.S. Agency for International Development, Washington, DC USA; 3 U.S. President’s Malaria Initiative, US Embassy Ouagadougou, Ouagadougou, Burkina Faso; 4 PMI VectorLink Project, Abt Associates Inc, 6130 Executive Blvd, Rockville, MD 20852 USA; 5 PMI VectorLink Burkina Faso, Abt Associates Inc, Ouagadougou, Burkina Faso; 6 Division of Parasitic Diseases and Malaria, Center for Global Health, Centers for Disease Control and Prevention, Atlanta, GA 30333 USA; 7 Centers for Disease Control and Prevention, U.S. President’s Malaria Initiative, Atlanta, GA USA

**Keywords:** Pyrethroid, Insecticide resistance, Piperonyl butoxide, Chlorfenapyr, *Anopheles gambiae*, Insecticide-treated net, Malaria, Burkina Faso

## Abstract

**Background:**

Pyrethroid resistance poses a major threat to the efficacy of insecticide-treated nets (ITNs) in Burkina Faso and throughout sub-Saharan Africa, particularly where resistance is present at high intensity. For such areas, there are alternative ITNs available, including the synergist piperonyl butoxide (PBO)-based ITNs and dual active ingredient ITNs such as Interceptor G2 (treated with chlorfenapyr and alpha-cypermethrin). Before deploying alternative ITNs on a large scale it is crucial to characterize the resistance profiles of primary malaria vector species for evidence-based decision making.

**Methods:**

Larvae from the predominant vector, *Anopheles gambiae *sensu lato (*s.l.*) were collected from 15 sites located throughout Burkina Faso and reared to adults for bioassays to assess insecticide resistance status. Resistance intensity assays were conducted using WHO tube tests to determine the level of resistance to pyrethroids commonly used on ITNs at 1×, 5 × and 10 × times the diagnostic dose. WHO tube tests were also used for PBO synergist bioassays with deltamethrin and permethrin. Bottle bioassays were conducted to determine susceptibility to chlorfenapyr at a dose of 100 µg/bottle.

**Results:**

WHO tube tests revealed high intensity resistance in *An. gambiae s.l.* to deltamethrin and alpha-cypermethrin in all sites tested. Resistance intensity to permethrin was either moderate or high in 13 sites. PBO pre-exposure followed by deltamethrin restored full susceptibility in one site and partially restored susceptibility in all but one of the remaining sites (often reaching mortality greater than 80%). PBO pre-exposure followed by permethrin partially restored susceptibility in 12 sites. There was no significant increase in permethrin mortality after PBO pre-exposure in Kampti, Karangasso-Vigué or Mangodara; while in Seguenega, Orodara and Bobo-Dioulasso there was a significant increase in mortality, but rates remained below 50%. Susceptibility to chlorfenapyr was confirmed in 14 sites.

**Conclusion:**

High pyrethroid resistance intensity in *An. gambiae s.l.* is widespread across Burkina Faso and may be a predictor of reduced pyrethroid ITN effectiveness. PBO + deltamethrin ITNs would likely provide greater control than pyrethroid nets. However, since susceptibility in bioassays was not restored in most sites following pre-exposure to PBO, Interceptor G2 may be a better long-term solution as susceptibility was recorded to chlorfenapyr in nearly all sites. This study provides evidence supporting the introduction of both Interceptor G2 nets and PBO nets, which were distributed in Burkina Faso in 2019 as part of a mass campaign.

**Supplementary Information:**

The online version contains supplementary material available at 10.1186/s12936-021-03936-3.

## Background

Burkina Faso relies on mass distribution of insecticide-treated nets (ITNs) every three years as the primary method of vector control, while indoor residual spraying (IRS) has been implemented in two or three districts per year since 2018 [1]. Millions of pyrethroid ITNs have been distributed in Burkina Faso during the past 20 years which, along with agricultural use of pyrethroids, has increased the selection pressure on malaria vector populations. Pyrethroid resistance was observed in *Anopheles gambiae *sensu lato (*s.l.*) populations for the first time in Côte d’Ivoire in 1993 [2], and within a few years was detected throughout all countries in West Africa, including Burkina Faso, and is now found across all of sub-Saharan Africa [3, 4]. The voltage-gated sodium channel gene *(Vgsc)*-L1014F mutation (also known as *kdr*-west) was assumed to be the main mechanism conferring resistance to pyrethroids in West Africa [5, 6], while in East Africa, a different amino acid substitution at the same locus known as *Vgsc*-L1014S (also known as *kdr*-east) had been detected [7]. Voltage-gated sodium channel gene mutations were first identified within *An. gambiae* populations [5, 6] across West Africa before it was detected within *Anopheles coluzzii* [8], most likely as a result of introgression, while its occurrence in wild *Anopheles arabiensis* populations was identified as an independent mutation [9]. While *Vgsc* mutations are widespread and contribute to phenotypic pyrethroid resistance, metabolic resistance mechanisms are generally more important drivers of high intensity pyrethroid resistance [10].

Pyrethroid resistance poses a threat to the efficacy of ITNs, particularly when resistance is present at high intensity [11, 12]. In Burkina Faso, pyrethroid resistance has been observed nationwide for several years and there are fears that pyrethroid ITNs may no longer provide the desired levels of individual and community protection [6]. While there are several localized studies in Burkina Faso that have described the genetic mutations involved in pyrethroid resistance, including *Vgsc*-1014F and 1014S mutations in association with N1575Y mutations and metabolic resistance patterns [13, 14], there is limited bioassay information regarding the relative improvement in mortality provided by piperonyl butoxide (PBO) synergists within wild *An. gambiae s.l.* populations. Reduced mosquito mortality by pyrethroid ITNs was reported in experimental hut trials in Benin in 2007 [15] and more recently in Burkina Faso [16] with free-flying *An. gambiae s.l.* In order to preserve the efficacy of ITNs and other insecticide-based control methods such as IRS, the World Health Organization (WHO) has developed a global plan for insecticide resistance management (GPIRM) [17]. Among the key elements are: (i) rotation of insecticides; (ii) mixtures of at least two different insecticides; (iii) alternating use of at least two insecticides from different classes; and, (iv) mosaic use of insecticides.

A limiting factor preventing implementation of these strategies has been the lack of alternative classes of insecticide for ITNs. However, in recent years several PBO synergist nets have received prequalification (PQ) listing by the WHO [18]. PBO is a synergist that acts by inhibiting metabolic enzymes within the mosquito, particularly cytochrome P450s that detoxify or sequester pyrethroids [19]. Although synergists do not typically act as insecticides, they can increase or restore the potency of insecticides by overcoming existing resistance mechanisms [19, 20]. As a result, PBO nets should produce an increased killing effect of malaria vectors where the major pyrethroid resistance mechanisms are due to increased oxidase activity. Two randomized control trials in Tanzania and Uganda have demonstrated a clinical benefit of PBO nets in areas of moderate pyrethroid resistance, with a reduction in malaria prevalence detected for at least six months after net distribution compared to conventional pyrethroid nets [21, 22].

A new ‘dual active ingredient’ net with two different active ingredients (AIs) is Interceptor® G2 (combining chlorfenapyr and alpha-cypermethrin), which received WHO PQ listing in 2018 [18]. Chlorfenapyr is a pyrrole compound with a non-neurotoxic mode of action which shows no cross resistance to existing pyrethroid resistance mechanisms [23, 24]. Experimental hut studies of Interceptor G2 have shown high efficacy and wash durability against pyrethroid resistant malaria vectors in Benin [25], Burkina Faso [26] and Côte d’Ivoire [27].

To guide National Malaria Control Programme (NMCP) decision-making regarding choice of ITN type, it is crucial to regularly gather insecticide susceptibility data for those insecticides used on ITNs, namely pyrethroids with and without PBO synergist, and chlorfenapyr. This study evaluated the level of pyrethroid resistance intensity in *An. gambiae s.l.* against the main pyrethroids used on ITNs followed by synergist assays to determine whether the synergist PBO restores susceptibility. In addition, the study determined susceptibility of *An*. *gambiae s.l.* to chlorfenapyr.

## Methods

### Study sites

**Fig. 1:**
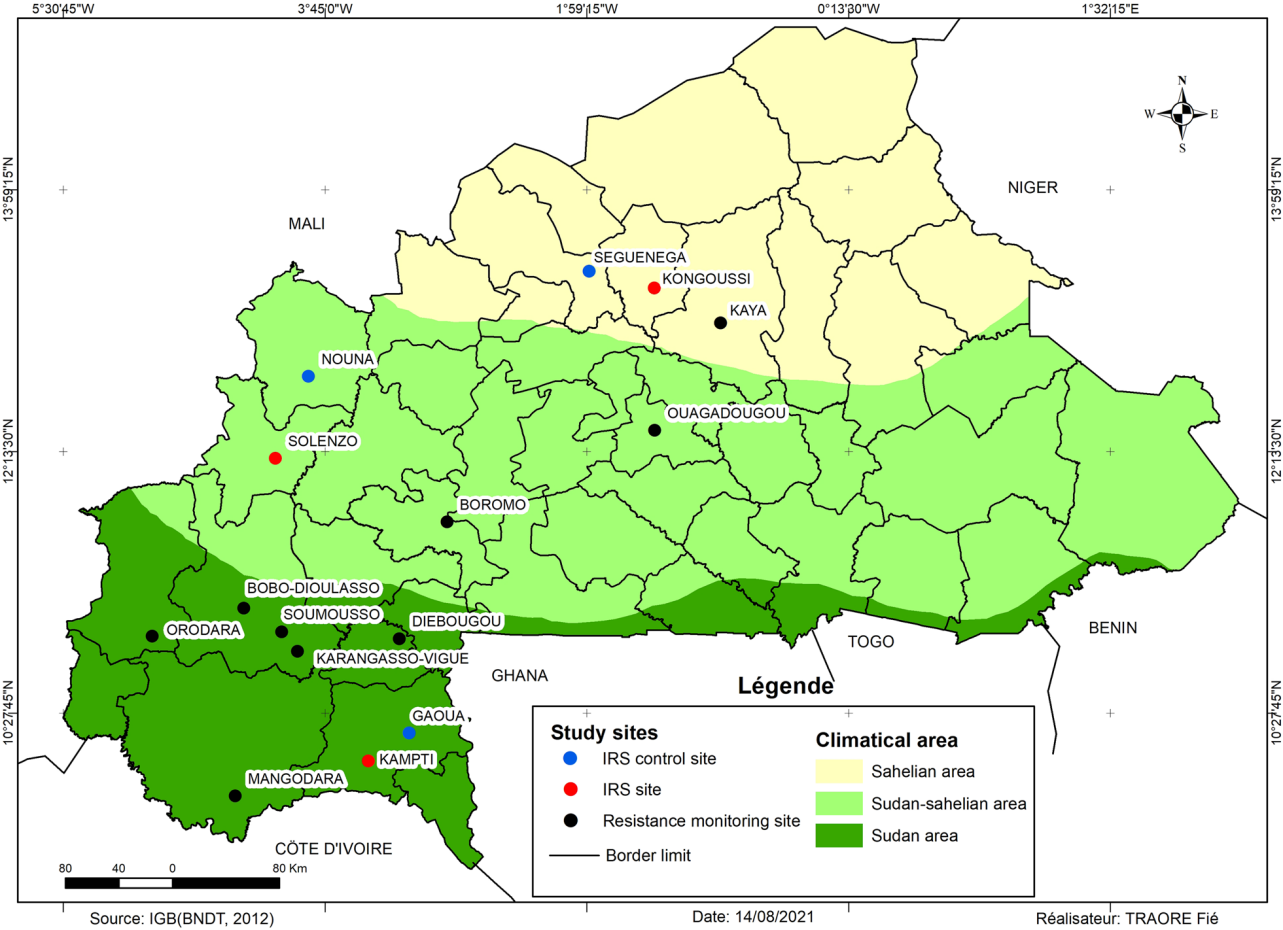
Location of 15 NMCP selected insecticide resistance monitoring sites where testing was conducted in 2019 (including 3 sites where IRS was conducted and 3 neighbouring unsprayed site)

The NMCP of Burkina Faso selected 21 sentinel sites for malaria vector insecticide resistance monitoring studies (Fig. 1). The sites were dispersed nationwide through the three eco-climatic zones of the country (Sahelian, Sudan-Sahelian, Sudan). For security reasons, it was not possible to monitor all 21 sites in 2019, especially sites located in the Sahelian zone and eastern part of country, therefore, the study covered 15 sites located in the three climatic areas, including IRS sites and neighbouring unsprayed control sites (Additional file 1). The Sudanian climatic zone covers the southwestern area (including Orodara, Bobo-Dioulasso, Soumousso, Karangasso-Vigué, Diébougou, Gaoua, Kampti, and Mangodara sites) and is the wettest in the country, with the mean annual rainfall averaging 1,000–1200 mm with most occurring in the rainy season extending from May to November, whereas the northern Sahelian zone (Kongoussi and Seguenega) is relatively dry, with less than 600 mm annual rainfall. In the central Sudano-Sahelian zone (Solenzo, Nouna, Boromo, Ouagadougou, and Kaya), the average yearly rainfall is 600–900 mm per year, with a shorter rainy season than in the southwest, extending from June to September 2019.

### Mosquito collections

Mosquito larval collections were carried out between August and October 2019 during the period of high malaria vector density. *Anopheles gambiae s.l.* larvae were collected from temporary water pools from all 15 sites before transport to the Research Institute of Health Sciences (IRSS) insectary and were reared to the adult stage prior to use in bioassays. Female *An. gambiae s.l.* were then used for insecticide susceptibility assays according to WHO procedures [28]. Species composition among the *An. gambiae* species complex was determined through PCR after completion of bioassay and is described below.

### Laboratory testing procedures

#### Pyrethroid insecticide susceptibility and resistance intensity tests using WHO tube tests

Three pyrethroid insecticides were used for susceptibility tests at their diagnostic concentrations: permethrin 0.75%, deltamethrin 0.05% and alpha-cypermethrin 0.05%. Pyrethroid resistance intensity was monitored in 14 locations (resistance intensity testing was not conducted in Bobo-Dioulasso) with alpha-cypermethrin, deltamethrin and permethrin papers treated at 1, 5 and 10 times the diagnostic concentration using WHO tubes. Prior to the field susceptibility assays, two impregnated papers with each insecticide were randomly selected from each box and tested against a laboratory-maintained susceptible strain of *An. gambiae* Kisumu to verify the quality of the paper. On average, batches of 20–25 sugar-fed female *An. gambiae s.l.* aged 3–5 days old were introduced into each WHO tube and exposed to the insecticide-treated paper for one hour [28] at 27 ± 2 °C temperature and 70 ± 5% relative humidity (Additional file 2). Four replicates were conducted, making a total of approximately 100 mosquitoes tested for each insecticide per site. Negative control bioassays were conducted for each test by exposing a total of 50 *An. gambiae s.l.* (two replicates of 25) using tubes lined with filter papers treated with silicone oil. Positive control bioassays were also conducted for each insecticide with an insectary colony of susceptible *An. gambiae* Kisumu (4 replicates per insecticide). The knock-down rates were recorded at 5, 10, 15, 20, 30, 40, 50 and 60 min after the start of exposure with mortality recorded 24 h after insecticide exposure [28]. All mosquitoes were held for 24 h in laboratory conditions at 27 ± 2 °C temperature and 70 ± 5% relative humidity to evaluate the mortality. Insecticide-treated papers were supplied by the WHO Collaborating Centre of Universiti Sains Malaysia (USM).

#### PBO synergist assays using WHO tube tests

Synergist bioassays were also conducted by exposing batches from 20 to 25 sugar-fed female *An. gambiae s.l.* in WHO tubes lined with PBO (4%)-impregnated papers for one hour before being transferred to a second tube with a pyrethroid insecticide (either permethrin (0.75%), deltamethrin (0.05%) or alpha-cypermethrin (0.05%)) impregnated paper for one hour (Additional file 3). The number of dead mosquitoes was recorded 24 h after the end of the exposure period at 27 ± 2 °C temperature and 70 ± 5% relative humidity. Negative controls consisted of two batches of around 20–25 mosquitoes exposed to PBO without subsequent exposure to pyrethroid insecticide and also silicone oil-treated papers. Each test consisted of four replicates. Synergist tests were conducted in all 15 sites with deltamethrin and permethrin but only five sites with alpha-cypermethrin due to insufficient mosquitoes in 10 sites during larval collections.

#### Chlorfenapyr susceptibility tests using CDC bottle bioassays

During the time of data collection there was no published guidance from WHO regarding chlorfenapyr susceptibility test procedures or diagnostic concentrations. Preliminary bottle bioassay testing by Center for Disease Control and Prevention (CDC) protocol established a tentative diagnostic dose of 100 µg/bottle. Therefore, 250-ml Wheaton bottles were treated in the IRSS laboratory using technical grade chlorfenapyr dissolved in acetone at a dosage of 100 µg/bottle. Mortality rates were assessed at 24, 48 and 72 h after exposure. Four replicates of 25 *An. gambiae s.l.* were conducted, making a total of approximately 100 mosquitoes tested per site. Tests were conducted during the daytime with effort made to keep testing and holding conditions within WHO guidelines of 27 ± 2 °C and relative humidity of 75 ± 10% [28].

#### Molecular assays to determine *Anopheles gambiae s.l.* species composition and characterization of resistance mutations

After the bioassay tests, all mosquitoes were placed individually in 1.5-ml tubes with silica gel before being stored in a − 20 °C freezer at IRSS until further laboratory analysis. A sub-set of approximately 50 female *An. gambiae s.l.* per site were tested by PCR to determine species composition. Genomic DNA of mosquitoes was extracted with 2% cetyl trimethyl ammonium bromide (2% CTAB). Species of the *An. gambiae* complex were identified by PCR as either *An. gambiae *sensu stricto (*s.s*.), *An. coluzzii* or *An. arabiensis* using the Sine 200X protocol of Santolamazza et al. [29]. Mutations involved in insecticide resistance were identified by PCR using the protocols of Martinez-Torres et al. [30] and Ranson et al. [7] for the voltage-gated sodium channel (*Vgsc*) L1014F and L1014S mutations.

### Data analysis

Data were entered into Microsoft Excel and analysed with STATA version 13.0 (College Station, TX 77,845, USA). WHO criteria were used to classify wild *An. gambiae* s.l. as ‘resistant’ if less than 90% mortality was observed, resistance needing confirmation if mortality was between 90–97% and susceptible if between 98–100% [28]. Resistance intensity was defined as being high resistance intensity if mortality at the 10 × dose was less than 98%, moderate intensity if less than 98% at the 5 × dose but greater than 98% at 10x, and low intensity resistance if mortality was greater than 98% at the 5 × dose [28].

The results of synergist assays were analysed by insecticide by comparing results of PBO plus pyrethroid *versus* pyrethroid only. The data were then interpreted following WHO criteria [28]:

- Complete restoration of susceptibility following pre-exposure to PBO (i.e. ≥ 98% mean mortality) implies that a monooxygenase-based resistance mechanism fully accounts for expression of the resistant phenotype in the test population.
- Partial restoration of susceptibility following pre-exposure to PBO (i.e. mean mortality in the PBO followed by insecticide samples is greater than mean mortality in the insecticide only samples but less than 98%) implies that a monooxygenase-based resistance mechanism only partially accounts for expression of the resistant phenotype and that other resistance mechanisms are likely to be present in the test population.
- No restoration of susceptibility following pre-exposure to PBO (mean mortality in the PBO followed by insecticide samples is equal to or lower than mean mortality in the insecticide only samples) implies that the resistance phenotype detected is not based on mono-oxgenase-mediated detoxification.

Descriptive statistics were used to calculate the mean mortality rates and differences between the average mortality rate. Mortality rates from bioassays were calculated with their 95% confidence interval (CI) and compared by ecological zone using Chi-squared test. The knockdown times for 50 and 95% of tested mosquitoes (KdT50 and KdT95) were calculated using a log time probit model [31] for mosquito exposure to PBO followed by deltamethrin and permethrin exposure. The KdT_50_ and KdT_95_ were estimated for sites located in Sudan areas where the mass coverage of PBO-pyrethroid ITNs were targeted. The genotypic frequencies of *Vgsc*1014F and 1014S in mosquito populations were compared to Hardy–Weinberg expectations using GenePOP software [32].

## Results

### Distribution of *Anopheles gambiae* species

**Fig. 2:**
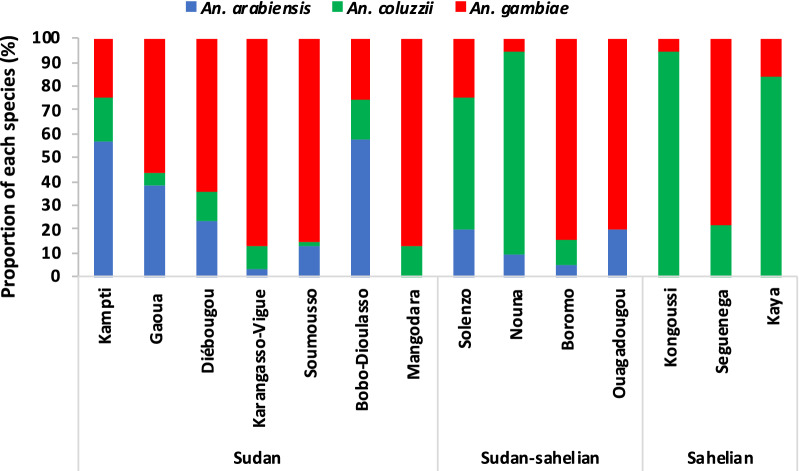
Members of the *Anopheles gambiae s.l.* present at each study sites (n≈50 females mosquitoes analysed per site)

Of 700 *An. gambiae s.l.* analysed by PCR, 51.8% were *An. gambiae s.s.*, 30.6% *An. coluzzii* and 17.6% *An. arabiensis*. *Anopheles gambiae s.s.* was the predominant species in the Sudan area (61.6%), except in Kampti and Bobo-Dioulasso where more than 50% were *An. arabiensis* (Fig. 2). In the Sudano-Sahelian zone *An. coluzzii* was more common in Nouna and Solenzo, where it was the dominant species (> 50%); however, in Boromo and Ouagadougou, more than 80% were *An. gambiae s.s*. *Anopheles coluzzii* was the predominant species in Sahelian area (67.3%). In all three eco-climatic zones, *An. gambiae s.s.* was found in sympatry with *An. coluzzii*. *Anopheles arabiensis* was found in relatively high proportions in the Sudanian zone at 27.4% (95%CI: 0.6–51.3).

### Pyrethroid susceptibility

**Fig. 3:**
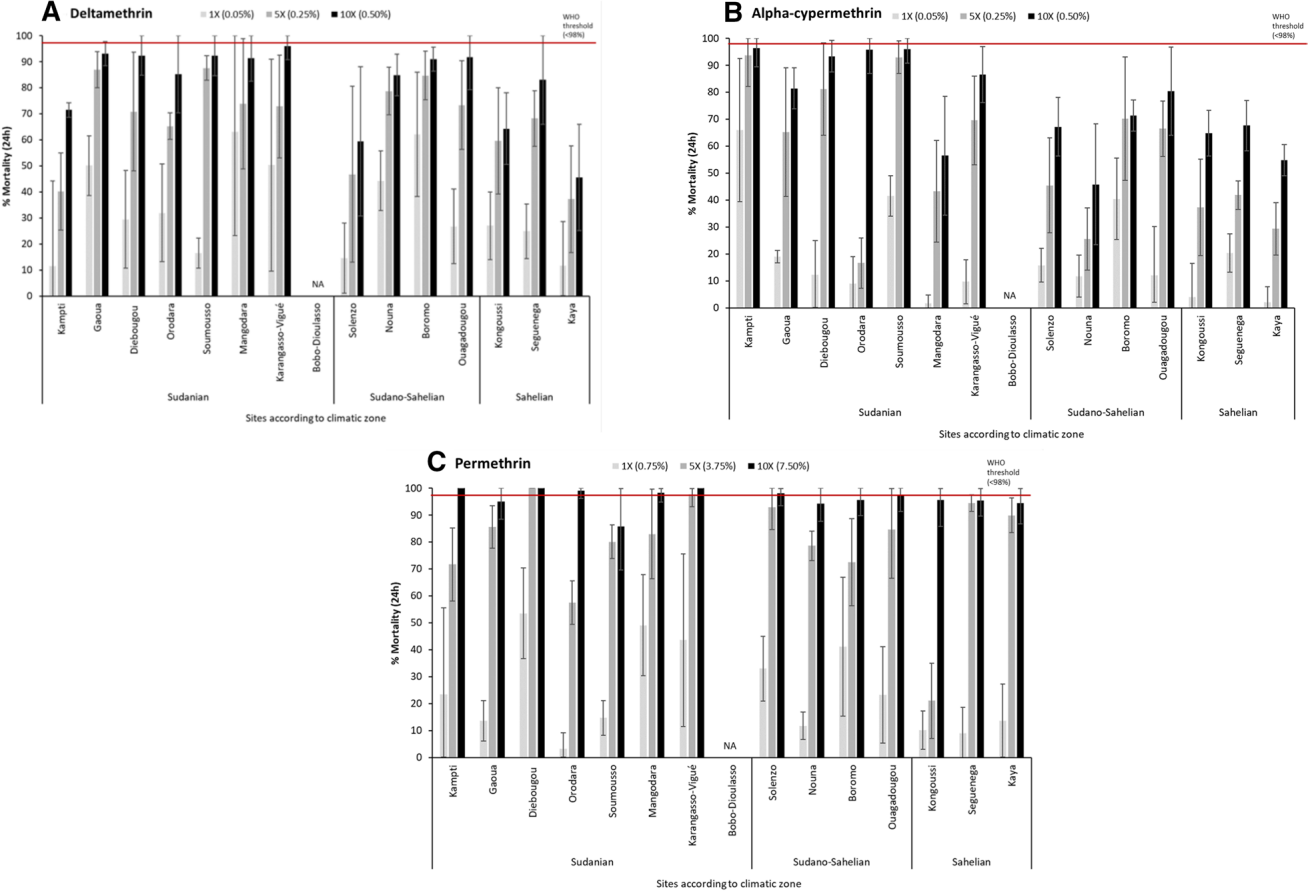
Percentage mortality (24 h) of *Anopheles gambiae s.l.* in WHO tube tests at 1, 5, and 10 times the diagnostic concentration of **A** deltamethrin (0.05%, 0.25%, 0.50%), **B** alpha-cypermethrin (0.05, 0.25, 0.50%) and **C** permethrin (0.75, 3.75, 7.50%) (n≈100 female mosquitoes per dose/insecticide)

WHO tube tests revealed that *An. gambiae s.l.* were resistant to the three pyrethroid insecticides tested, with mortality rates less than 70% at all sites for deltamethrin, permethrin and alpha-cypermethrin diagnostic doses (Fig. 3). Mean mortality rates across the 14 sites were 33.2% for deltamethrin, 24.5% for permethrin and 19.0% for alpha-cypermethrin (Fig. 3). There was particularly low mortality in Kaya for all three pyrethroids: 11.8% (95% CI:0.1–28.6) mortality for deltamethrin, 13.6% (95% CI:0.1–27.2) for permethrin and 2.0% (95% CI:0.1–7.8) for alpha-cypermethrin. There were many examples of sites having very low mortality rates for individual pyrethroid insecticides. The susceptible *An. gambiae* Kisumu strain exhibited 100% mortality to all three pyrethroids, therefore confirming that insecticide-treated papers were dosed correctly. Control mortality was less than 5% in all bioassays.

### Pyrethroid resistance intensity

The results showed high resistance intensity in all 14 sites to deltamethrin and alpha-cypermethrin (mortality less than 98% at the 10 × dose) with *An. gambiae s.l.* (Fig. 3A, B). Mortality rates varied between 45.6% in Kaya to 96.0% in Karangasso-Vigue at the 10 × dose of deltamethrin (Fig. 3A). The results with alpha-cypermethrin at the 10 × dose showed mortality rates between 45.8% in Nouna and 96.3% in Kampti (Fig. 3B). The resistance intensity of *An. gambiae s.l.* to permethrin was more varied with eight sites classified as high intensity, five as moderate and one with low resistance intensity (Diebougou) (Fig. 3C).

### PBO plus pyrethroid synergist assays

**Fig. 4:**
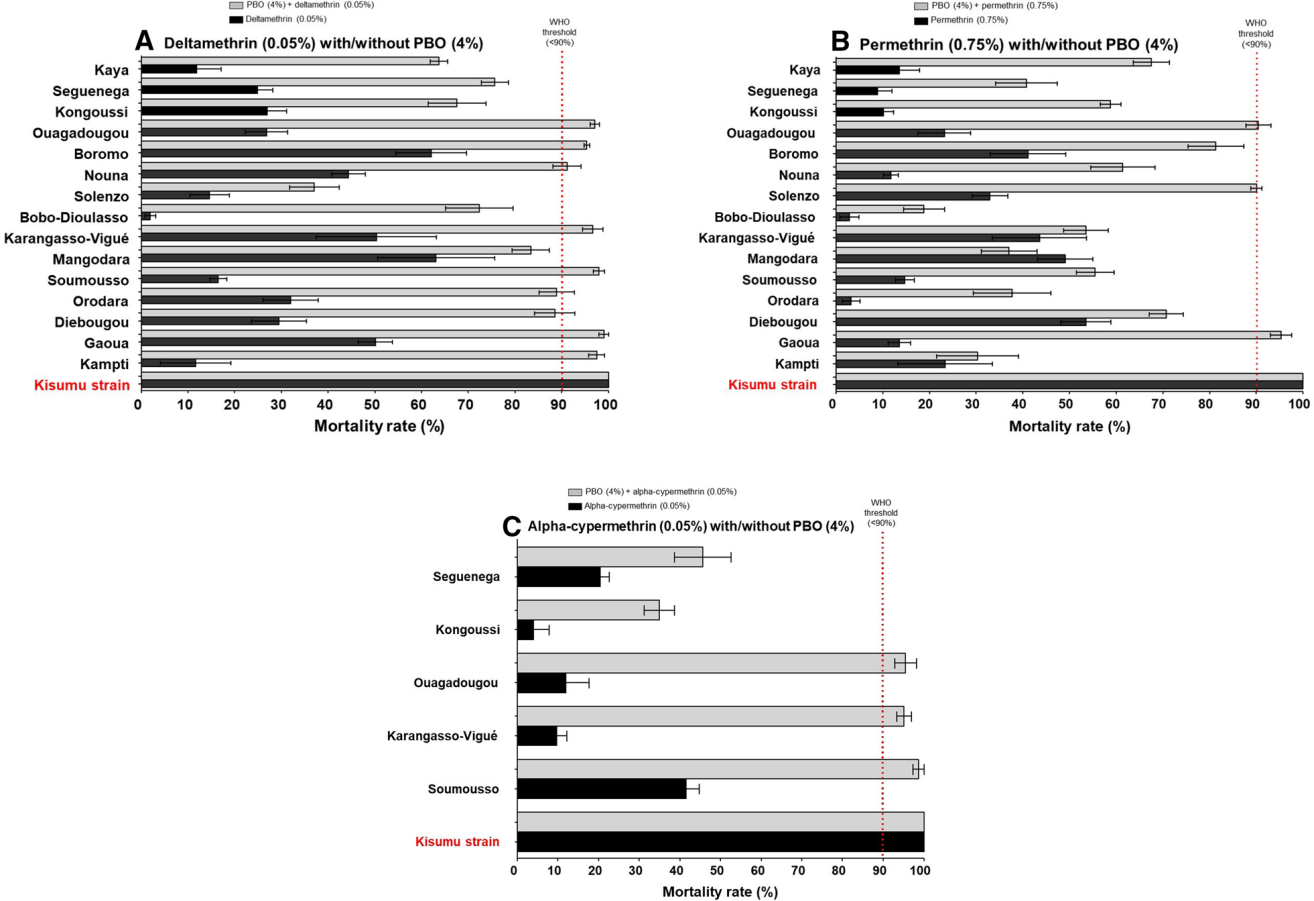
Percentage mortality (24 h) of *Anopheles gambiae s.l.* in WHO tube tests with **A** deltamethrin (0.05%) with/without PBO (4%) and **B** permethrin (0.75%) with/without PBO (4%) **C** alpha-cypermethrin (0.05%) with/without PBO (4%)

Pre-exposure to PBO (4%) followed by a pyrethroid insecticide significantly improved mortality rates in all sites for deltamethrin (P < 0.05) although there was great variation between sites (Fig. 4A). PBO pre-exposure followed by deltamethrin restored full susceptibility only in Gaoua but partially restored susceptibility in the remaining sites (often reaching mortality greater than 80%) except Solenzo where the mortality remained below 50%.

PBO pre-exposure followed by permethrin did not restore full susceptibility in any site, but did partially restore susceptibility in 12 sites from the 14 sites tested. However, there was no significant increase in mortality in Kampti, Karangasso-Vigué or Mangodara. While in Seguenega, Orodara and Bobo-Dioulasso there was a significant increase in mortality, but rates remained below 50% (Fig. 4B). Synergist tests with alpha-cypermethrin were only conducted in five sites. Mortality of *An. gambiae s.l.* reached more than 90% after pre-exposure to PBO followed by alpha-cypermethrin in three of five sites (Ouagadougou, Karangasso-Vigué and Soumousso) (Fig. 4C). In the remaining two sites of Kongoussi and Seguenega there was partial restoration of susceptibility, but mortality rates did not reach 50%.

The analysis of knockdown times (KDT) showed that the pre-exposure of *An. gambiae s.l.* to PBO reduced both the KDT_50_ and KDT_95_ (time needed to achieve 50 and 95% mosquito knockdown) compared to those mosquitoes only exposed to deltamethrin or permethrin. The KDT_50_ and KDT_95_ times with deltamethrin after exposure to PBO were 2 to 3 times faster compared to those obtained with deltamethrin only (Table 1). KDT_50_ and KDT_95_ values for PBO after exposure to permethrin showed similar trends with two-fold faster knockdown than permethrin only (Table 1).

**Table 1:** Time to knockdown (in minutes) for 50% and 95% (KDT_50_ and KDT_95_) of *Anopheles gambiae s.l*. from the Sudanian area following exposure to pyrethroids only and PBO plus pyrethroid

Climatical area	Insecticides	KDT_50_ (min)	[95% IC] KDT_50_	KDT_95_ (min)	[95% IC] KDT95
Sudan	Deltamethrin	48.61	[45.43–52.59]	146.48	[123.23–182.75]
Sudan	PBO + deltamethrin	22.97	[22.06–23.89]	48.46	[45.31–52.41]
Sudan	Permethrin	154.21	[111.65–262.32]	1051.22	[520.39–3427.81]
Sudan	PBO + permethrin	78.29	[66.69–97.07]	530.84	[348.65–955.87]

### Chlorfenapyr susceptibility

**Fig. 5:**
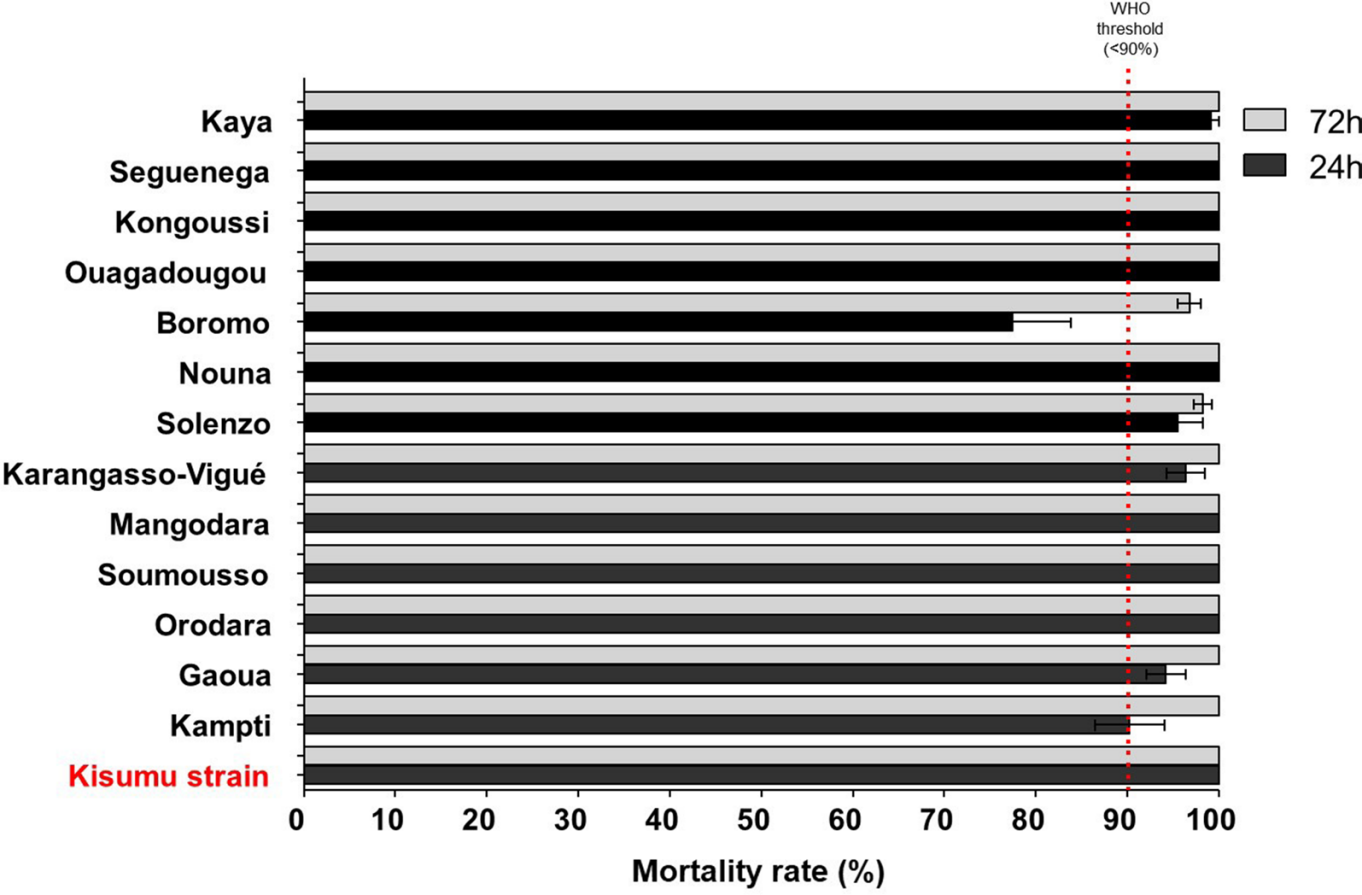
Percentage mortality (24 and 72 h) of *Anopheles gambiae s.l.* in susceptibility tests with chlorfenapyr in bottle bioassays at a dose of 100 µg AI/bottle

The results of susceptibility tests performed with *An. gambiae s.l.* against chlorfenapyr at 100 µg/bottle revealed high mortality (98–100%) 24 h after exposure in most sites. Susceptibility to chlorfenapyr was confirmed in 14 sites using 72 h mortality data, with Boromo the only site where mortality was less than 98% (Fig. 5; Additional file 4).

### Frequency of the *Vgsc*-L1014F and 1014S mutations

The *Vgsc*-L1014F mutation was observed at varying frequencies between sites and by mosquito species (Table 2). This mutation was found at a higher frequency within *An. gambiae s.s.* populations with allele frequencies varying from 0.53 in Boromo to 0.98 in Soumousso (for sites where more than 20 samples were tested). The mean frequency within *An. gambiae s.s.* (n = 310) across all sites was 0.71 for *Vgsc*-L1014F and 0.13 for *Vgsc*-L1014S, with 5% of this species having both alleles present.

**Table 2:** Allele frequency of the *Vgsc*-L1014F and *-*L1014S mutations in *Anopheles gambiae s.l.* populations in 2019

Species	Sites	N	L1014L	L1014F	L1014S	L1014F	L1014L	L1014L	Allele frequency	
			L1014L	L1014F	L1014S	L1014S	L1014F	L1014S	L1014F	L1014S
*An. gambiae s.s*	Kampti	13	0	11	2	0	0	0	0.85	0.15
	Gaoua	27	0	21	3	1	1	1	0.81	0.15
	Solenzo	9	1	5	2	0	1	0	0.61	0.22
	Nouna	2	0	1	1	0	0	0	0.50	0.50
	Kongoussi	2	0	0	0	1	0	1	0.25	0.50
	Seguenega	46	2	9	3	11	14	7	0.34	0.14
	Orodara	44	2	38	0	0	4	0	0.91	0
	Soumousso	42	1	41	0	0	0	0	0.98	0
	Ouagadougou	40	1	37	0	0	0	2	0.93	0.03
	Boromo	34	7	14	1	3	5	4	0.53	0.13
	Mangodara	43	7	27	3	0	6	0	0.70	0.07
	Kaya	8	2	0	0	1	4	1	0.25	0.06
	Total	310	23	204	15	17	35	16	0.71	0.13
*An. coluzzii*	Kampti	9	1	2	0	1	4	1	0.50	0.11
	Gaoua	3	0	2	0		0	1	0.67	0.17
	Solenzo	26	3	10	1	7	4	1	0.60	0.19
	Nouna	43	7	16	6	4	7	3	0.50	0.22
	Kongoussi	47	2	16	2	8	19	0	0.63	0.13
	Seguenega	10	0	4	0	2	3	1	0.65	0.15
	Orodara	2	0	0	2	0	0	0	0	1.00
	Soumousso	1	0	1	0	0	0	0	1.00	0
	Ouagadougou	0	0	0	0	0	0	0	NA	NA
	Boromo	10	1	3	0	0	3	3	0.45	0.15
	Mangodara	7	0	2	5	0	0	0	0.29	0.71
	Kaya	42	10	8	3	1	16	5	0.39	0.14
	Total	200	24	64	19	23	56	15	0.52	0.19
*An. arabiensis*	Kampti	28	0	11	0	11	1	5	0.60	0.29
	Gaoua	19	0	9	1	5	1	3	0.63	0.26
	Solenzo	9	6	2	0	1	0	0	0.28	0.06
	Nouna	5	2	0	0	0	2	1	0.20	0.10
	Kongoussi	0	0	0	0	0	0	0	NA	NA
	Seguenega	0	0	0	0	0	0	0	NA	NA
	Orodara	4	0	4	0	0	0	0	1.00	0
	Soumousso	6	4	0	2	0	0	0	0	0.33
	Ouagadougou	10	0	10	0	0	0	0	1.00	0
	Boromo	3	0	3	0	0	0	0	1.00	0
	Mangodara	0	0	0	0	0	0	0	NA	NA
	Kaya	0	0	0	0	0	0	0	NA	NA
	Total	84	12	39	3	17	4	9	0.59	0.19

In *An. coluzzii* (n = 200), the mean *Vgsc*-L1014F frequency was lower than that in *An. gambiae s.s.* at 0.52. The mean *Vgsc*-L1014S frequency was 0.19 in *An. coluzzii*. More surprisingly, both allele mutations were also present in *An. arabiensis* populations at a similar frequency to *An. coluzzii*, with a mean of 0.52 for *Vgsc*-L1014F and 0.19 for *Vgsc*-L1014S. Comparing by climatic zones, the *Vgsc*-L1014F mutation was higher in Sudanian and Sudano-Sahelian areas (χ2 = 12.91, df = 1.311, P < 0.0001) than that in Sahelian sites.

The L1014S mutation is now widespread throughout the country within *An. gambiae s.s.* and *An. coluzzii* and was found in six sites within *An. arabiensis* populations, although the frequency was relatively low in all sites with 0.29 for *An. arabiensis* in Kampti being the highest frequency for any site (where > 10 samples were tested).

Significant deviations from Hardy–Weinberg expectations were observed in *An. gambiae* populations from Kongoussi, Seguenega, Ouagadougou, Boromo, and Kaya, and *An*. *coluzzii* populations from Kampti, Gaoua, Solenzo, Boromo, and Seguenega. This was due to strong heterozygote deficits.

## Discussion

This study have demonstrated for the first time, using standardized WHO protocols [28], that high intensity pyrethroid resistance is not geographically confined to areas of intense agriculture but is widespread throughout Burkina Faso. Given that the primary malaria vector control strategy in Burkina Faso is nationwide universal coverage with ITNs, of particular concern is the high intensity of resistance to deltamethrin and alpha-cypermethrin documented at every site tested. Pyrethroid resistance intensity is an important measure that may indicate the potential for reduced effectiveness of pyrethroid ITNs. WHO suggest that “confirmed levels of resistance, especially at ten times the discriminating concentration may indicate or predict operational control failure and highlight a particularly urgent need to develop an appropriate resistance management strategy” [28]. High intensity resistance was previously only documented in intensive agricultural areas, such as Vallée du Kou, an area of concentrated rice cultivation 25 km from Bobo-Dioulasso, where deltamethrin resistance up to 1,000 fold was reported in *An. coluzzii* in 2013 compared to the susceptible Kisumu strain of *An. gambiae* [16].

To supplement universal coverage with pyrethroid ITNs, which are deployed via mass campaigns every three years, US President’s Malaria Initiative (PMI)-funded IRS with non-pyrethroid insecticides (organophosphates and neonicotinoids) has also been conducted annually since 2018 in three high burden districts (Kampti, Solenzo, Kongoussi) in accordance with the WHO GPIRM recommendations. However, the relatively high cost of IRS makes the prospect of national use of this strategy low and PBO or dual active ingredient nets are likely to be the primary response for control of pyrethroid-resistant malaria vectors. A barrier limiting the purchase of PBO nets by NMCPs and donors is that PBO ITNs cost approximately 40% more than equivalent pyrethroid ITNs [33]. Therefore, it is important to target distribution to locations where PBO synergist bioassays indicate full or partial restoration of susceptibility to pyrethroids. Toé et al*.* [34] showed in Vallée du Kou and Tengrela (southwestern Burkina Faso) evidence of synergism but pre-exposure to PBO did not fully restore susceptibility to deltamethrin or permethrin in bioassays or in experimental hut studies. Bayili et al. [35] also showed in experimental hut studies in Vallée du Kou that deltamethrin plus PBO nets provided additional benefit over the equivalent pyrethroid-only LLINs, but mortality and blood-feeding inhibition rates were relatively low. While these studies provided important information, they were limited in geographical scope. Results from study showed that mosquito mortality rates nationwide significantly increased when *An. gambiae s.l.* were pre-exposed to PBO in association with permethrin, deltamethrin and alpha-cypermethrin even in areas of high resistance intensity in western part of country (Mangodara, Gaoua, Kampti). In all sites, pre-exposure to PBO also reduced the mean KDT compared to exposure to only pyrethroids. While randomized controlled trials have shown the benefit of PBO nets in Tanzania [21] and Uganda [22] in areas of moderate pyrethroid resistance, it is not clear what level of increased mortality in susceptibility bioassays is required to correlate with epidemiological impact. WHO provide limited guidance, stating that the added benefit of pyrethroid PBO nets compared to pyrethroid-only LLINs is expected to be the greatest where pyrethroid resistance is at “intermediate levels”, where mosquito mortality after exposure to a pyrethroid insecticide in WHO test kits ranges from 10 to 80%, but do not state what level of mortality increase is biologically significant [36]. The most recent PMI Malaria Operational Plan Guidance recommends that PBO nets only be considered in areas where PBO increases pyrethroid susceptibility by at least 10% (absolute terms) [37]. Based on results from study, PBO plus deltamethrin ITNs are the most promising option in Burkina Faso as greater than 70% mortality was reached from bioassays in 12 of 15 sites, with susceptibility restored in one site and a greater than 10% absolute increase in mortality in all sites.

Laboratory analysis showed that the frequency of the *Vgsc*-L101F mutation is moderate or high in *An. gambiae s.s.*, *An. coluzzii* and *An. arabiensis.* The frequency of the *Vgsc*-L1014S mutation is also increasing in frequency compared to prior years, particularly in *An. gambiae s.s.* [38]. Surprising results found also were the similar allele mutations frequencies in both species *An. coluzzi* and *An. arabiensis*. Indeed, this finding could be explained by the colonization of *An. coluzzi* breeding sites by *An. arabiensis* species particularly in urban sites exposed to insecticides pressure. Previous microarray analysis of *An. coluzzii* from Vallée du Kou has implicated several P450 genes including CYP6P3 and CYP6Z2 [39]. Similarly, Toé et al. [34] found CYP6Z2 and CYP6Z3 the most highly overexpressed genes in Vallée du Kou and Tengrela. This suggests that there are complex underlying resistance mechanisms involved, including target site resistance, metabolic resistance mechanisms and there may be other mechanisms not yet detected, such as increased cuticle thickness. It is important for further studies to be conducted over a wider geographical distribution to do molecular and genetic investigations to have a better understanding of the specific cytochrome P450 enzymes and mono-oxygenases.

While there is a good prospect that PBO plus deltamethrin ITNs will provide greater control than pyrethroid ITNs in most locations of Burkina Faso in the short term, there are already examples in parts of Côte d’Ivoire [40] and Mozambique [41] where PBO nets no longer provide additional benefit due to the high intensity of pyrethroid resistance and the resistance mechanisms present. Interceptor G2 may be a longer term alternative for improved control of pyrethroid-resistant mosquitoes as chlorfenapyr has a completely different mode of action and works through oxidative phosphorylation of the cell mitochondria [42]. Large-scale testing of susceptibility to chlorfenapyr at the interim diagnostic dose of 100 µg/bottle was conducted for the first time in Burkina Faso and showed susceptibility in nearly all sites. Experimental hut trials in Burkina Faso [26], Benin [25] and Côte d’Ivoire [27] have shown higher vector mortality with Interceptor G2 even when washed 20 times to simulate field use. The results of randomized controlled trials being conducted in Benin and Tanzania to determine the epidemiological impact of Interceptor G2 nets compared to pyrethroid nets are eagerly awaited before widespread distribution can be recommended by WHO. In the interim, as part of the ‘New Nets Project’, pilot distribution of PBO and Interceptor G2 nets was conducted in parts of Burkina Faso in 2019 [43]. Districts were selected to receive PBO and Interceptor G2 nets based on the results of insecticide resistance monitoring, along with other operational criteria. Given the increased costs of PBO and Interceptor G2 nets it is important to continue monitoring their lifetime durability including physical integrity, residual insecticidal efficacy and chemical retention over three years.

## Conclusion

High pyrethroid resistance intensity in malaria vector species is widespread across Burkina Faso and may be a predictor of reduced pyrethroid ITN effectiveness. Pre-exposure to the synergist PBO significantly increased vector mortality, resulting in partial or full restoration of susceptibility in most sites. PBO plus deltamethrin ITNs should be prioritized for distribution in Burkina Faso as this combination provided the highest levels of mortality increase. However, susceptibility was not restored in most sites and dual active ingredient nets, such as Interceptor G,2 may be a better long-term solution.

## Supplementary Information

**Additional file 1.** Study sites distributed in three eco-climatical areas with different agricultural practices.

**Additional file 2.** Temperature and humidity conditions of WHO susceptibility and CDC bottle bioassays.

**Additional file 3.** Synergist assays using pyrethroid insecticides and piperonyl butoxide data.

**Additional file 4.** CDC bottle assays using 100ug chlorfenapyr data.

## Data Availability

All data generated or analysed during this study are included in this published article and additional files.

## Abbreviations

CDC: Center for Disease Control and Prevention
CI: Confidence interval
GPIRM: Global plan for insecticide resistance management
IRSS: Institut de Recherche en Sciences de la Santé
IRS: Indoor residual spraying
ITNs: Insecticide-treated nets
*Kdr*: Knockdown resistance
KdT50: Knockdown times for 50%
KdT95: Knockdown times for 95%
LLINs: Long-lasting insecticidal nets
NMCP: National Malaria Control Programme
PBO: Piperonyl butoxide
PMI: US President’s Malaria Initiative
s.l.: Sensu lato
s.s.: Sensu stricto
USM: Universiti Sains Malaysia
*Vgsc*: Voltage-gated sodium channel gene
*Vgsc*-L1014F: *kdr*-West
*Vgsc*-L1014S: *kdr*-East
WHO: World Health Organization
