# Supplementary material for: Evidence supporting deployment of next generation insecticide treated nets in Burkina Faso: bioassays with either chlorfenapyr or piperonyl butoxide increase mortality of pyrethroid-resistant Anopheles gambiae
Source: Malar J. 2021 Oct 18;20:406. doi: 10.1186/s12936-021-03936-3 (PMC8524873; doi:10.1186/s12936-021-03936-3)
Supplement: Supplementary file 1 — Additional file 1. Study sites distributed in three eco-climatical areas with different agricultural practices. [file 12936_2021_3936_MOESM1_ESM.pdf]

Additional file

Table SI. Study sites distributed in three eco-climatical areas with different agricultural practices

Climatic Area	Region	Sentinel Site	Co-ordinates	Social environment	Agricultural practice
Sudanian	Sud-Ouest	Kampti	10°7′N, 3°27'W	Sub-urban	Cereals, cotton
		Gaoua	10°40’N, 3°15’W	Sub-urban	Cereals, cotton since 1960s
		Diebougou	10°95’N, 3°24’W	Sub-urban	Cereals, cotton since 1960s, USAID IRS site 2010-2012
	Cascades	Mangodara	9°54′N, 4°21′W	Rural	Cereals, cotton
	Hauts-Bassins	Orodara	11°00’N, 4°91’W	Sub-urban	Fruit, cotton since 1960s
		Bobo-Dioulasso	11°11’N, 4°17’W	Urban	Vegetables, domestic use of insecticides
		Soumousso	11°01’N, 4°02W	Rural	Cotton, semi-permanent swamp
		Karangasso-Vigué	10°52’N, 3°56’W	Rural	Rice, vegetables, cotton
Sudano-sahelian	Boucle du Mouhoun	Solenzo	12°11'N, 4°05'W	Rural	Cotton, cereals
		Nouna	12°37’N, 3°55’W	Sub-urban	Cotton, cereals
		Boromo	11°75’N, 2°92’W	Sub-urban	Cotton, cereals
	Centre	Ouagadougou	12°22’ N, 1°31’W	Urban	Vegetables, domestic use of insecticides
Sahelian	Centre- Nord	Kongoussi	13°19′ N, 1°32′W	Sub-urban	Cereals
		Seguenega	13°15′N, 1°58′W	Rural	Cereals, vegetables (permanent water source)
	Sahel	Kaya	13°05’N, 1°05’W	Sub-urban	Cereals
